# Genetic subtyping and phylogenetic analysis of HA and NA from avian influenza virus in wild birds from Peru reveals unique features among circulating strains in America

**DOI:** 10.1371/journal.pone.0268957

**Published:** 2022-06-07

**Authors:** Gina R. Castro-Sanguinetti, Paulo Vitor Marques Simas, Ana Paola Apaza-Chiara, Jose Alonso Callupe-Leyva, Juan Alexander Rondon-Espinoza, Cesar M. Gavidia, Juan Anderson More-Bayona, Rosa Isabel Gonzalez Veliz, Vikram N. Vakharia, Maria Eliana Icochea

**Affiliations:** 1 Laboratory of Avian Pathology, Faculty of Veterinary Medicine, Universidad Nacional Mayor de San Marcos, Lima, Lima, Peru; 2 Laboratory of Epidemiology and Veterinary Economy, Faculty of Veterinary Medicine, Universidad Nacional Mayor de San Marcos, Lima, Lima, Peru; 3 Laboratory of Microbiology and Parasitology, Virology Section, Faculty of Veterinary Medicine, Universidad Nacional Mayor de San Marcos, Lima, Lima, Peru; 4 Institute of Marine & Environmental Technology, University of Maryland, Baltimore County, Baltimore, MD, United States of America; Foshan University, CHINA

## Abstract

Avian influenza virus (AIV) represents a major concern with productive implications in poultry systems but it is also a zoonotic agent that possesses an intrinsic pandemic risk. AIV is an enveloped, negative-sense and single-stranded RNA virus with a segmented genome. The eight genomic segments, comprising the whole genome, encode for eleven proteins. Within these proteins, Hemagglutinin (HA) and Neuraminidase (NA) are the most relevant for studies of evolution and pathogenesis considering their role in viral replication, and have also been used for classification purposes. Migratory birds are the main hosts and play a pivotal role in viral evolution and dissemination due to their migratory routes that comprise large regions worldwide. Altogether, viral and reservoir factors contribute to the emergence of avian influenza viruses with novel features and pathogenic potentials. The study aimed to conduct surveillance of AIVs in wild birds from Peru. A multi-site screening of feces of migratory birds was performed to isolate viruses and to characterize the whole genome sequences, especially the genes coding for HA and NA proteins. Four-hundred-twenty-one (421) fecal samples, collected between March 2019 and March 2020 in Lima, were obtained from 21 species of wild birds. From these, we isolated five AIV from whimbrel, kelp gull, Franklin’s gulls and Mallard, which were of low pathogenicity, including four subtypes as H6N8, H13N6, H6N2 and H2N6. Genetic analysis of HA and NA genes revealed novel features in these viruses and phylogenetic analysis exhibited a close relationship with those identified in North America (US and Canada). Furthermore, H2N6 isolate presented a NA sequence with higher genetic relationship to Chilean isolates. These results highlight that the geographical factor is of major relevance in the evolution of AIV, suggesting that AIV circulating in Peru might represent a new site for the emergence of reassortant AIVs.

## Introduction

Avian influenza viruses (AIV) infect animals and humans [1] and thus are of major relevance under the one health context. They are enveloped viruses belonging to Alphainfluenzavirus from *Orthomyxoviridae* family [2]. These negative sense and single-stranded RNA viruses present a genome that is fragmented into eight segments which encode for eleven proteins. These proteins possess individual function or form complexes during viral replication. The mechanisms underlying AIVs evolution, comprising antigenic shift and drift, point mutations, reassortments and others, are induced mainly by their segmented genome and the receptor specificity, including a wide host range into the animal kingdom [3].

Hemagglutinin (HA) and neuraminidase (NA) are structural proteins responsible for attachment and viral release, respectively. These proteins play a fundamental role in AIV replication. In addition, HA and NA have also been used for viral classification into subtypes. Thus, eighteen HAs and eleven NAs have been described to date [4]. From these, sixteen types of HA (H1-16) and nine of NA (N1-9) were identified in avian species, including wild and migratory birds from *Anseriformes* and *Charadriiformes* orders [5]. Other two subtypes, H17N10 and H18N11, have been identified in bats from Guatemala and Peru [6].

Most AIV subtypes have been classified as low pathogenicity avian influenza viruses (LPAIV) and generally induce minimal impact in the health of domestic and wild birds, although there have been reports of sporadic cases in humans related to H5, H6, H7, H9 and H10 avian subtypes [7]. Animal reservoir carrying these viruses spread AIV on the ecosystem, facilitating an effective transmission between vulnerable hosts. Furthermore, genetic changes in relevant proteins, such as HA and NA, can turn them into highly pathogenic avian influenza viruses (HPAIV) with high consequences for other species [8].

Infections by HPAIV have a major social and economic impact in poultry because it can result in massive deaths in the farms, and has a potential for causing disease in humans [9, 10]. In addition, global population expansion has provided new opportunities for spillover events. Altogether, active epidemiological surveillance under a one health approach is essential to develop novel strategies that will allow us to implement measures in a concerted manner before epidemics begins [11]. Hence, it is essential to get a better understanding of viral circulation patterns in our ecosystem, providing fundamental information for AIV risk assessment and detecting probable hotspots of novel AIV emergence [12].

Waterfowl has been considered the main natural reservoir for all AIV subtypes representing an important source for transmission to other species [13]. Moreover, Peru has a central location within the migration route of wild birds in Americas, and therefore is of relevance for viral spreading [14]. Hence, our major objective was the isolation and genetic subtyping and characterization of the AIV circulating in wild birds in Peru, using the HA and NA genetic information, and establishing relationships to other isolates previously detected in America.

## Materials and methods

### Experimental design and samples

A descriptive, qualitative, cross-sectional study of active epidemiological surveillance for avian influenza in animal reservoir in the Coast of Lima (Peru) was conducted in the period of March 2019 and March 2020. Sampling was carried out targeting different population of waterfowl species belonging to *Charadriiformes* and *Anseriformes* orders. This included several residents and migratory waterfowl in Peruvian territory. Fecal samples were collected from the ground using swabs and Universal Transport Media (UTM) (Copan Diagnostics Inc., USA) in cool chain boxes. Samples were taken in 5 natural resting areas for waterfowl in Lima (Puerto Viejo 76.7026578°W 12.5714006°S, Pantanos de Villa 76.9949416°W 12.2065568°S, Albufera de Medio Mundo 77.6626868°W 10.9353162°S, Laguna La Encantada 77.5522327°W 11.1364147°S and Poza de La Arenilla 77.1590185°W 12.0721548°S). Avian species were determined under close visual observation and photographic record, and classified according to phenotypic characteristics. Thus, fecal samples were taken only when the avian species could be determined.

Samples were transported to the Avian Pathology Laboratory of the School of Veterinary Medicine, Universidad Nacional Mayor de San Marcos (Lima). Fecal samples were processed and clarified by centrifugation (8000 x g for 10min) and supernatants were filtered through 0.22-micron syringe filters. Filtered samples were then pooled according to species, collection date and geographical location into 4 to 5 samples per pool, supplemented with 1000 U/mL Penicillin G, 1 mg/mL Streptomycin and 10 μg/mL amphotericin B, and incubated at 4°C for 1 hour prior to inoculation into embryonated eggs.

### Virus isolation in embryonated chicken eggs

Viral isolation was performed by inoculation of 0.2 mL of pooled samples in ten-day-old specific-pathogen-free (SPF) embryonated eggs through allantoic sac route in five replicates. This process was followed by incubation at 37°C for 120 hours [15]. Embryo survival was checked daily. Allantoic fluid from each sample was tested by hemagglutination assay as initial screening. Positive samples were titrated using the same technique by serially diluting with two-fold volume of PBS (pH 7.4) and then incubating with equal volume of 1% chicken red blood cells (RBC). After positivity evidence, the positive pool had their samples evaluated individually [16].

### Viral detection

Rapid immunochromatographic assay (QuickVue Influenza A+B Test, US) was used for rapid identification of Influenza A virus in allantoic fluid samples that tested positive for hemagglutination test. For confirmation, we used a PCR-based method to amplify the NP gene from the viral genomic material. Thus, the viral RNA was extracted from 140 μL of allantoic fluid using QIAamp Viral RNA Mini Kit (Qiagen, US), according to manufacturer instructions. This was followed by a step of reverse transcription using GoScript™ Reverse Transcription System kit (Promega, USA). In this step, we used the primer described by Hoffman et al. [17] (Uni-12 5’-AGCAAAAGCAGG-3’) that allows amplification of all influenza A segments. NP gene was detected by PCR using GoTaq® G2 Green Master Mix (Promega, USA) and primers described by Wright et al. (1995) [18] Anp-1 (5’- ATCACTCACTGAGTGACATC-3’) and Anp-2 (5’-CCTCCAGTTTTCTTAGGATC-3’) for 306-bp. The thermal cycling conditions were 94°C for 3 minutes; 35 cycles of 94°C for 30s, 58°C for 30s and 72°C for 60s; and 72°C for 5 minutes. PCR products were electrophoresed on 1.5% agarose gels and visualized by ethidium bromide staining.

### Next generation sequencing (NGS)

First strand cDNA was prepared using SuperScript™ III First-Strand Synthesis kit (Invitrogen TM), following manufacturer recommendations and using the primer FR20RV (5’-GCCGGAGCTCTGCAGATATC-3’) prior to library preparation [19, 20]. The libraries were prepared using Nextera XT method and sequenced on a MiSeq platform using paired 150 base read chemistry (Cambridge Technologies, Worthington, MN, USA).

### Assembly and annotation

The raw data was submitted for end trimming using *Trimmomatic*, a flexible tool for Illumina NGS data [21] and quality control evaluation based on Phred quality scores [22]. Reads that presented a high Phred score were submitted for *de novo* genome assembly (DISCOVAR) [23]. Segments that were not properly assembled were submitted to a new round using PRICE [24]. The annotation was performed using “Influenza Virus Resource” tool, from the “National Center for Biotechnology Information”–NCBI, available at https://www.ncbi.nlm.nih.gov/genomes/FLU/annotation/. After annotation, the segments were edited manually.

### Sanger’s sequencing

For some isolates, complete nucleotide sequences of the 5’-end and 3’-end non-coding and coding regions of few gene segments were not obtained by NGS. Therefore, primer pairs were designed to amplify the non-coding and coding sequences of these gene segments by RT-PCR, as described earlier [25, 26]. The resulting amplicons were purified and DNA was directly sequenced or cloned into a pGEM-T vector, and the plasmid DNA was sequenced using Sanger’s dideoxy chain termination method. (Institute of Marine and Environmental Technology, Baltimore, MD, USA).

Thus, complete nucleotide sequences for the whole genome of Peruvians AIVs were attained, namely PE-01 to PE-5, and the data was deposited into the GenBank.

### NA and HA subtyping and phylogenetic analysis

Following sequencing, we focused on the subtyping of AIV isolated based on the HA and NA genetic analysis. Each HA and NA segment assembled was submitted to genetic characterization using MEGA [27]. Criteria for databank construction considered the evolutionary pattern of AIV in the ecosystem (host, space and time). The coding region of HA and NA from all AIV subtypes from America available in the webpage of National Center for Biotechnology Information (NCBI), Influenza Virus Genome Set (https://www.ncbi.nlm.nih.gov/genomes/FLU/Database/nph-select.cgi?go=genomeset) were included and sequences that presented degenerated nucleotide were excluded. The alignment was conducted using MAFFT online service (https://mafft.cbrc.jp/alignment/server/) according to default parameters and the aligned sequences were posteriorly manually edited using BioEdit 7.2 software (https://bioedit.software.informer.com/7.2/). Phylogenetic analysis was performed using MEGA X (https://academic.oup.com/mbe/article/35/6/1547/4990887) determining the best evolution model by using the jModelTest2 software; clustering was obtained using the maximum-likelihood (ML) method under General Time Reversible gamma distribution and proportion of invariable sites parameters and 1,000 replicates of bootstrap.

## Results

To survey the presence of AIVs in wild birds from Peru, 421 fecal samples were inoculated in SPF chicken embryos and tested by hemagglutination assay (HA), which was performed by mixing equal volume of allantoic fluid and chicken red blood cells (1:1 dilution). Out of these, eight samples tested positive by HA. Further screening of all eight samples by immuno-chromatography and by RT-PCR amplification of the NP gene evidenced only five positive samples of AIVs (Table 1, S1 Fig).

**Table 1:** Status of samples tested positives by hemagglutination test followed by confirmation using rapid antigen and PCR tests for influenza A virus.

Samples	HA test (1:1 dilution)	Immunochromatography	PCR (NP gene)
1	**+**	**+**	**+**
2	**+**	**+**	**+**
3	**+**	**+**	**+**
4	**+**	**+**	**+**
5	**+**	**+**	**+**
6	**+**	**-**	**-**
7	**+**	**-**	**-**
8	**+**	**-**	**-**

From five AIV samples collected from the wetlands; two were from Poza de la Arenilla, two from Pantanos de Villa and one from La Encantada, all located in Lima province. Two positive samples were isolated from two franklin gulls in Poza de la Arenilla; one from a kelp gull and one from a whimbrel in Pantanos de Villa; and one from a mallard in La Encantada. A summary of these data, including general information about species and location is presented in Table 2.

**Table 2:** Twenty-one different avian species identified as possible reservoir of avian influenza virus in Lima Metropolitan Region, Peru, Pacific flyway, South America.

Common Name	Number of samples	HA positives	Location	ID
**American Oystercatcher**	26	0	-	-
**Belcher’s Gull **	20	0	-	-
**Black skimmer**	34	0	-	-
**Black Vulture**	2	0	-	-
**Black-crowned night heron**	3	0	-	-
**Blackish Oystercatcher**	4	0	-	-
**Common moorhen**	38	0	-	-
**Franklin’s gull **	54	2	Poza de la Arenilla, Callao 77.1595302°W 12.0717358°S	PE-03 PE-04
**Grey-headed Gull**	13	0	-	-
**Kelp gull**	2	1	Pantanos de Villa, Chorrillos 76.9949416°W 12.2065568°S	PE-02
**Killdeer**	8	0	-	-
**Mallard**	10	1	La Encantada, Huacho 77.5944682°W	PE-05
**Many-colored Rush-Tyrant**	2	0	-	-
**Neotropic cormorant**	91	0	-	-
**Peruvian Pelican**	37	0	-	-
**Pied-billed grebe**	2	0	-	-
**Snowy egret**	15	0	-	-
**Spotted sandpiper**	37	0	-	-
**Turkey Vulture**	1	0	-	-
**Whimbrel**	2	1	Pantanos de Villa, Chorrillos 76.9949416°W 12.2065568°S	PE-01
**White-cheeked Pintail**	20	0	-	-

Following detection of these AIV isolates, we performed the whole genome sequencing (WGS), which allowed us to characterize and identify close genetic relatives for each viral segment. Here, we focused on the annotation of HA and NA which reveal that our PE isolates correspond to the following subtypes: H6N8 (PE-01), H2N6 (PE-02), H6N2 (PE-03), H13N6 (PE-04) and H6N2 (PE-05) commonly found in wild birds. The information related to accession numbers and the closest relatives for each gene segments is presented in Table 3.

**Table 3:** Whole genome sequences of AIV isolates obtained in Peru and their closest relatives.

Sample/Subtype	Sampling date	Segments	GenBank Accession Number		Origin and Subtype of Reference Strain
			Peruvian Strain	Reference Strain	
**PE-01/H6N8**	May 5^th^, 2019	PB2	OL601968	MK237638	A/blue-winged teal/Illinois/17OS2553/2017 –H6N1
		PB1	OL601969	MH764210	A/ring-billed gull/Minnesota/CLMNAI1197/2017 –mixed
		PA	OL601970	OL355025 and MH764242	A/Mallard/Peru/M6/2019 –H2N6 and A/ring-billed gull/Minnesota/CLMNAI1501/2017
		HA	OL366799	MK236883	A/American green-winged teal/Missouri/17OS3048/2017 –H6N1
		NP	OL366800	MT566304	A/Mallard/Ohio/17OS1691/2017 –H3N8
		NA	OL366801	MH936480	A/blue-winged teal/Alberta/460/2017 –mixed
		MP	OL366802	MN253773	A/Mallard/Maine/AH0063771S.7.A/2015 –H11N1
		NS	OL366803	MN254207.1	A/Mallard/Minnesota/AH0031714S.8.A/2015 –H3N8
**PE-02/H2N6**	December 27^th^, 2019	PB2	OL355023	CY235256	A/mallard/Idaho/UGAI16-1941/2016 –mixed
		PB1	OL355024	MG266062	A/blue-winged teal/Wyoming/AH0099021/2016 –H7N9
		PA	OL355025	OL601970 and MH764242	A/Whimbrel/Peru/M5/2019 –H6N8 and A/ring-billed gull/Minnesota/CLMNAI1501/2017 –H13N2
		HA	OL355026	KY644183	A/blackish oystercatcher/Chile/C6534/2016 –H2N2
		NP	OL355027	MT566304	A/Mallard/Ohio/17OS1691/2017 –H3N8
		NA	OL355028	MK424190	A/Meleagris gallopavo/Valparaiso/10_2_10/2017 –H7N6
		MP	OL355029	MK071455	A/yellow-billed teal/Argentina/CIP112-1227/2016 –H4N6
		NS	OL355030	MH932199	A/blue-winged Teal/Alberta/459/2017 –H3N8
**PE-03/H6N2**	December 13^th^, 2019	PB2	OL355032	MK237300	A/bufflehead/Wisconsin/17OS4051/2017 –H1N1
		PB1	OL355033	CY206625	A/blue-winged teal/Texas/UGAI14-3363/2014 –H4N6
		PA	OL355034	MK236731	A/mallard/Ohio/17OS5632/2017 –H10N7
		HA	OL355035	MK236883	A/American green-winged teal/Missouri/17OS3048/2017 –H6N1
		NP	OL355036	MG280371	A/scoter/Maryland/16OS2529/2016 –H10N7
		NA	OL355037	MH499219	A/Franklin’s gull/Chile/C10242/2016 –H6N2
		MP	OL355038	MN253732	A/Blue-winged Teal/Minnesota/AH0031755S.7.A/2015 –H3N8
		NS	OL355039	MK345974	A/ruddy turnstone/Delaware Bay/190/2018 –H2N7
**PE-04/H13N6**	October 17^th^, 2019	PB2	OL355040	MH764127	A/ring-billed gull/Minnesota/CLMNAI0808/2017 –H13N8
		PB1	OL355041	MT624324	A/Mallard duck/Alberta/216/2019 –H6N5
		PA	OL355042	MH764181	A/ring-billed gull/Minnesota/CLMNAI0955/2017 –H13N8
		HA	OL355043	MH764042	A/ring-billed gull/Minnesota/OPMNAI0817/2017 –H13N8
		NP	OL355044	MH764179	A/ring-billed gull/Minnesota/CLMNAI0955/2017 –H13N8
		NA	OL355045	MH764203	A/ring-billed gull/Minnesota/CLMNAI1197/2017 –mixed
		MP	OL355046	OL367084 and MH763860	A/Franklins gull/Peru/M9/2019 –H6N2 and A/ring-billed gull/Minnesota/OPMNAI0816/2017 –H13N8
		NS	OL355047	MH764257	A/ring-billed gull/Minnesota/CLMNAI1539/2017 –H13N6
**PE-05/H6N2**	December 19^th^, 2019	PB2	OL602181	MT421768	A/northern shoveler/Maryland/UGAI16-5592/2016 –H11N9
		PB1	OL602182	CY235271	A/mallard/Idaho/UGAI16-1943/2016 –H3N8
		PA	OL367080	MK995693	A/Northern Shoveler/California/D1715696/2017 –H5N3
		HA	OL367081	MK236677	A/American wigeon/Missouri/17OS4599/2017 –H6N1
		NP	OL367082	MK995691	A/Northern Shoveler/California/D1715696/2017 –H5N3
		NA	OL367083	MK237026	A/mallard/Wisconsin/17OS3655/2017 –H3N2
		MP	OL367084	OL355046 and MH763860	A/Kelp gull/Peru/M8/2019 –H13N6 and A/ring-billed gull/Minnesota/OPMNAI0816/2017 –H13N8
		NS	OL367085	MH764257	A/ring-billed gull/Minnesota/CLMNAI1539/2017 –H13N6

**Fig 1:**
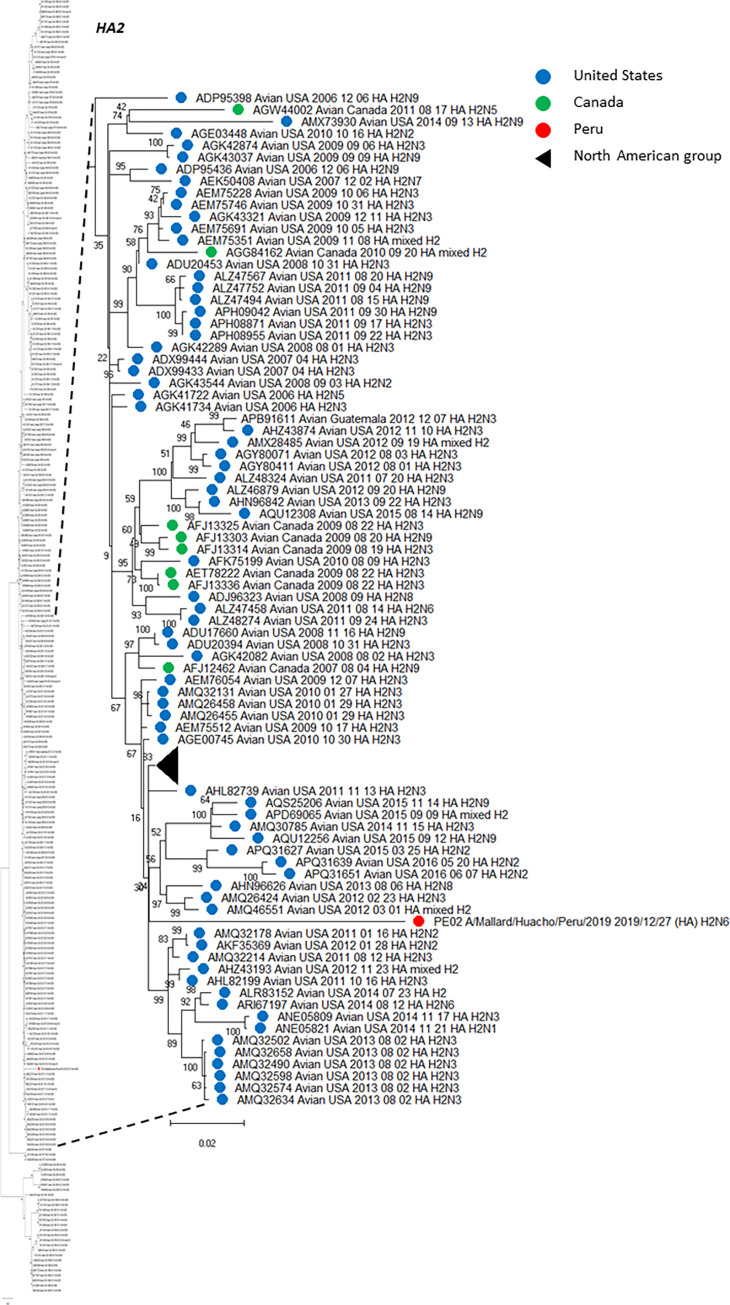
Phylogenetic tree of HA subtype H2 of an isolate detected in a kelp gull in Peru, and others detected in America. The tree was generated using a maximum likelihood method with 1000 replicates of bootstrap using GTR+G+I as nucleotide substitution model. They were included in the tree with the Peruvian strain (PE-02) and 256 sequences of complete coding region of HA subtype H2 that was detected in America through all time. A subtree of the complete phylogenetic analysis, including the isolate identified in our study is shown. H2 sequences from United states isolates (blue), Canada (green) and Peru (red) are deployed. A collapsed group of North American H2 sequences is shown in black triangle. The complete phylogenetic tree is shown at the side for reference.

**Fig 2:**
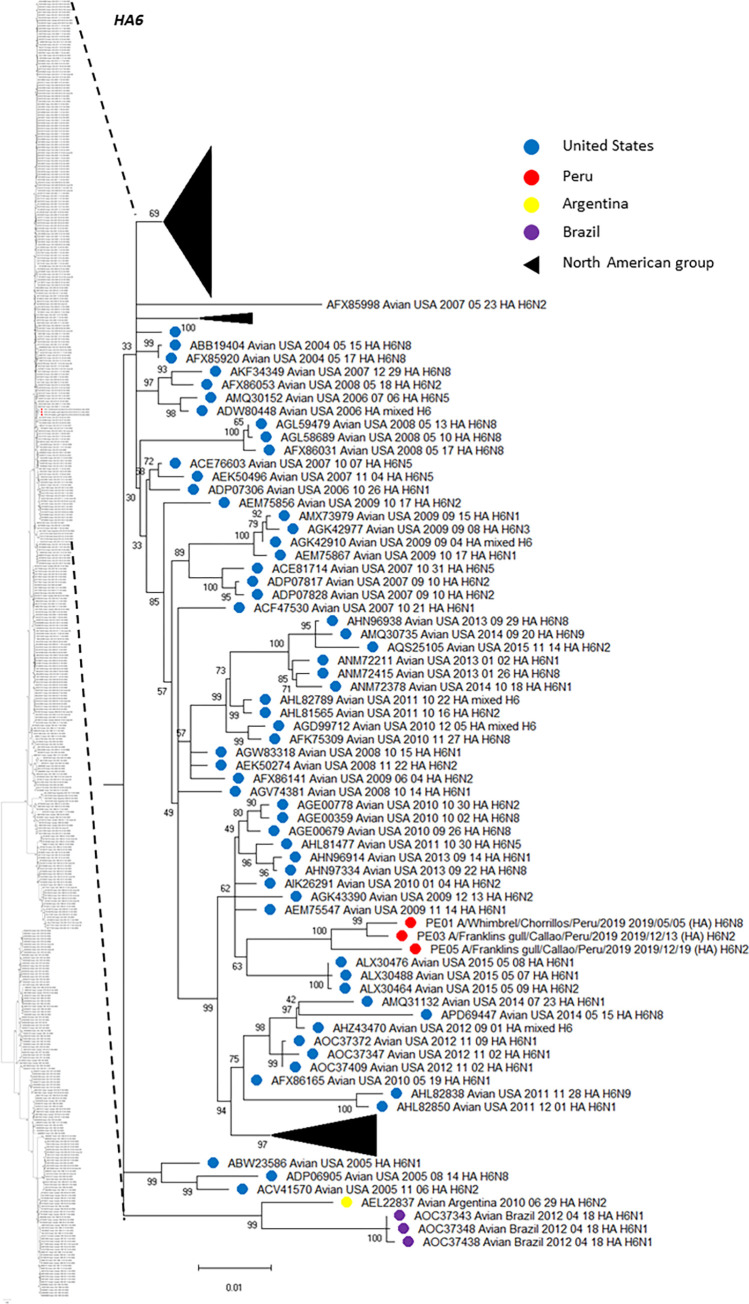
Phylogenetic tree of HA subtype H6 of three isolates detected in whimbrel and franklin gull in Peru, and others detected in America. The tree was generated using a maximum likelihood method with 1000 replicates of bootstrap using GTR+G+I as nucleotide substitution model. They were included in the tree with the Peruvian strains (PE-01, PE-03 and PE-05) and 477 sequences of complete coding region of HA subtype H6 that was detected in America through all time. A subtree of the complete phylogenetic analysis, including those isolates identified in our study is shown. H6 sequences from United states isolates (blue), Argentina (yellow), Brazil (purple) and Peru (red) are deployed. Collapsed groups of North American H6 sequences is shown in black triangle. The complete phylogenetic tree is shown at the side for reference.

**Fig 3:**
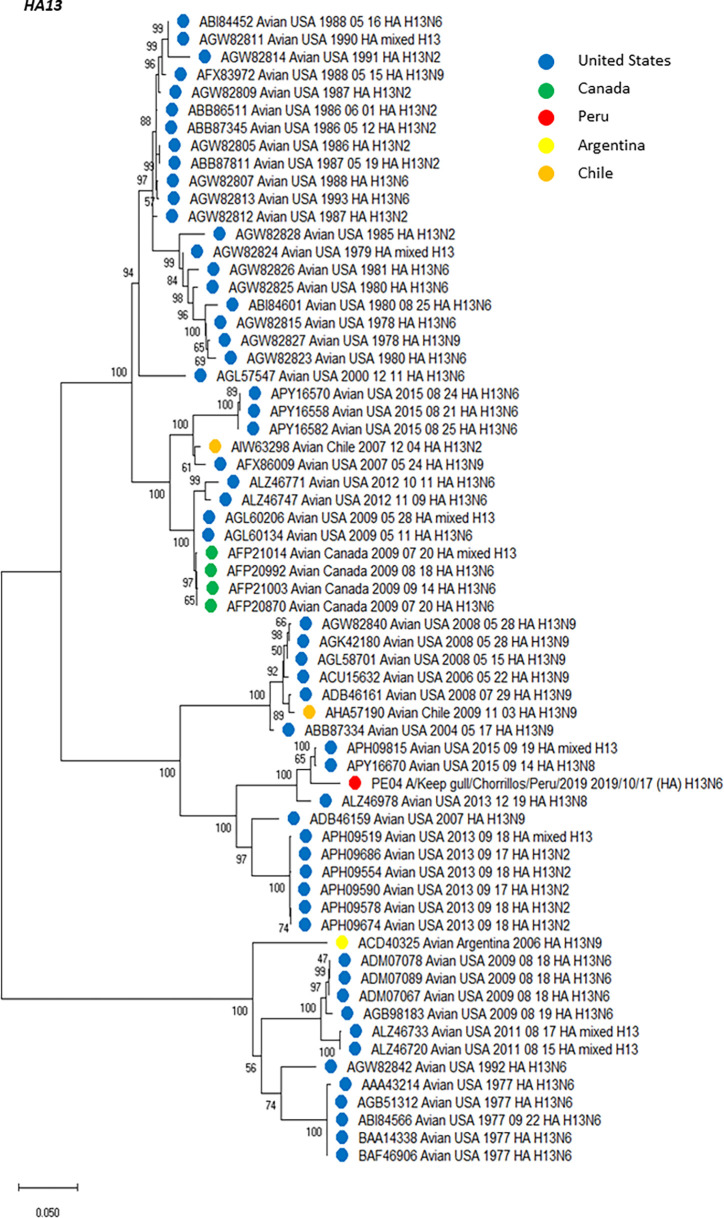
Phylogenetic tree of HA subtype H13 of an isolate detected in a kelp gull in Peru, and others detected in America. The tree was generated using a maximum likelihood method with 1000 replicates of bootstrap using GTR+G+I as nucleotide substitution model. They were included in the tree with the Peruvian strain (PE-04) and 64 sequences of complete coding region of HA subtype H13 that was detected in America through all time. H13 sequences from United States (blue), Canada (green), Argentina (yellow), Chile (orange) and Peru (red) are deployed.

An amino acid analysis was conducted and the list of amino acid substitutions for each HA and NA segments, including additional information is presented in Table 4 and Table 5, respectively. Using HA sequences, we deployed multiple phylogenetic trees, including those HA genetic information from virus subtypes that have been circulating in America. Using Mega 11, we created subtree versions of the complete phylogenetic analysis, which are shown in Figs 1 (H2), 2 (H6) and 3 (H13). Thus, H2 (PE-02) has emerged within a north American clade including isolates from 2011–2015 (Fig 1). All H6 genes (PE-01, PE-03 and PE-05) share a common ancestor and formed a sister group with another monophyletic clade formed by three isolates from US identified in 2015 (ALX30476, H6N1; ALX30488, H6N1 and ALX30464, H6N2) (Fig 2). The H13 (PE-04) sequence was more similar with a mixed strain from USA/2015 and a strain H13N8 identified in the US in 2017 (US/Minnesota/2017) with a 99.47% of amino acid identity (Fig 3). The phylogenetic tree showed a monophyletic group between our isolate with other two isolates from US identified in 2015, a mixed strain APH09815 and a strain H13N8 APY16670. Interestingly, we found multiple amino acid changes compared to their closest relatives. Thus, H2 (PE-02) has multiple changes which had the larger number of changes among our isolates and were also evidenced in the phylogenetic tree since that branch was more dissimilar than others in that group. Other HAs have also shown some amino acid changes but in lower proportions. In contrast, one of our H6N2 isolates (PE-03) did not evidence changes compared to its closest relative.

**Table 4:** Amino acid substitutions in the Hemagglutinin protein of AIV isolated from wild birds in Peru.

STRAIN		AMINO ACID SUBSTITUTIONS	IDENTITY (%)	SOURCE		SUBTYPE	
Peruvian	Best similar			Peruvian	Species	Peruvian	Highest identity
**PE01**	AWV92994.1	114 (A → V) 153–154 (DG → YN) 281 (V → I) 283 (R → K)	561/566 (99.12)	Fecal sample; Chorrillos-Peru; Whimbrel; May 5^th^, 2019	Feces after hatch year; ruddy turnstone, New Jersey-USA; May 26^th^, 2010.	H6N8	H6N1
**PE02**	QGT49557.1	59 (N → S) 81 (N → E) 157 (Q → R) 161–162 (IY → VS) 164 (G → N) 173 (T → V) 175 (A → K) 185 (K → Q) 199 (T → A) 203 (E → T) 249 (P → T) 273 (I → R) 323 (N → D) 358 (I → T) 404–405 (KA → EV) 417 (I → L) 456 (R → K) 491 (I → V) 510 (T → N)	541/562 (96.26)	Fecal sample; Huacho-Peru; Mallard; December 27^th^, 2019	Cloaca of *Bucephala clangula* (common goldeneye), Michigan-USA; December 9^th^, 2018.	H2N6	H2N1
**PE03**	AFX86165.1	-	566/566 (100)	Fecal sample; Callao-Peru; Franklin’s gull; December 13^th^, 2019	Canada goose; Delaware Bay-USA; May 19^th^, 2010.	H6N2	H6N1
**PE04**	AXO65592.1	231 (I → T) 385 (K → R) 402 (D → N)	562/565 (99.47)	Fecal sample; Chorrillos-Peru; Kelp gull; October 17^th^, 2019	Ring-billed gull; hatch year; Cass country, Minnesota-USA; July 18^th^, 2017.	H13N6	H13N8
**PE05**	QDX47360.1	04 (V → I) 275 (K → T)	564/566 (99.65)	Feces; Callao-Peru; Franklin’s gull; October 17^th^, 2019	Oropharyngeal/cloacal swab of blue-winged teal; Florida-USA; 2016.	H6N2	-

**Table 5:** Amino acid substitutions in the Neuraminidase protein of AIV isolated from wild birds in Peru.

STRAIN		AMINO ACID SUBSTITUTIONS	IDENTITY (%)	SOURCE		SUBTYPE	
Peruvian	Best similar			Peruvian	Species	Peruvian	Best similar
**PE01**	AVN99905.1	57 (I → V) 377 (K → R)	468/470 (99.57)	Fecal sample; Chorrillos-Peru; Whimbrel; May 5^th^, 2019	Wild bird feces; New Jersey-USA; May 14^th^, 2017	H6N8	H5N8
**PE02**	AYJ18735.1	44 (T → I) 75 (I → V) 79–80 (GK → ET) 172 (A → T) 213 (A → V) 241 (I → V) 262 (K → R) 305 (T → I) 426 (I → V)	460/470 (97.87)	Fecal sample; Huacho-Peru; Mallard; December 27^th^, 2019	Feces of *Meleagris gallopavo*; Chile; 2017	H2N6	H7N6
**PE03**	AXF49015.1	20 (V → I) 72 (I → T) 249 (K → R) 338 (K → R) 415 (K → R) 418 (V → I)	453/459 (98.69) 469 (cover query 98)	Fecal sample; Callao-Peru; Franklin’s gull; December 13^th^, 2019	Franklin’s gull; Chile; March 19^th^, 2016	H6N2	H6N2
**PE04**	AXO65346.1	43 (L → P) 59 (T → I)	468/470 (99.57)	Feces of Kelp gull; Chorrillos-Peru; October 17^th^, 2019	Ring-billed gull; Hatch year; Big Stone county, Minnesota-USA; July 26^th^, 2017	H13N6	H13N6
**PE05**	APQ31926.1	50 (A → V) 306 (D → N)	456/458 (99.56) 469 (cover query 96)	Feces of Franklin’s gull; Callao-Peru; October 17^th^, 2019	*Anas strepera;* Arkansas-USA; December 18^th^, 2015	H6N2	H5N2

**Fig 4:**
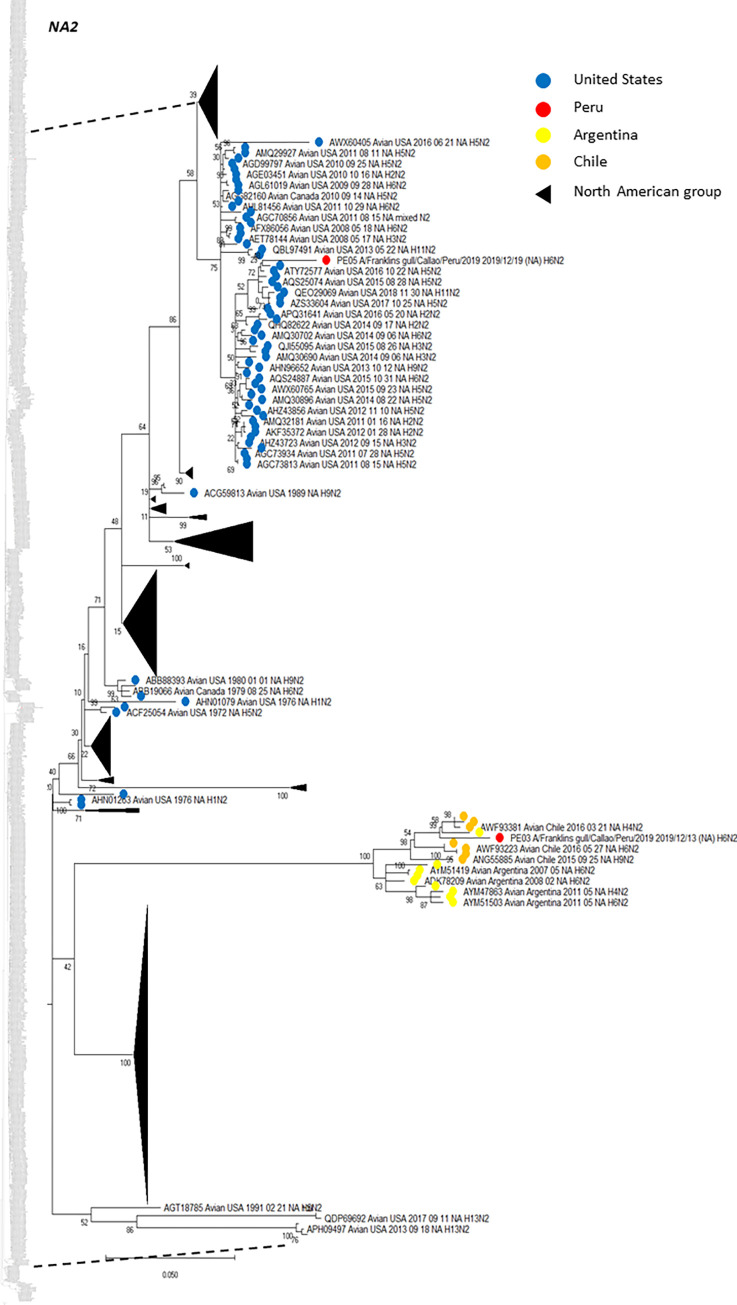
Phylogenetic tree of NA subtype N2 of isolates detected in two franklins gulls in Peru, and others detected in America. The tree was generated using a maximum likelihood method with 1000 replicates of bootstrap using GTR+G+I as nucleotide substitution model. They were included in the tree with the Peruvian strains (PE-03 and PE-05) and 1020 sequences of complete coding region of NA subtype N2 that was detected in America through all time. A subtree of the complete phylogenetic analysis, including isolates identified in our study is shown. N2 sequences from United States (blue), Argentina (yellow), Chile (orange) and Peru (red) are deployed. Collapsed groups of North American N2 sequences are shown in black triangles. The complete phylogenetic tree is shown at the side for reference.

**Fig 5:**
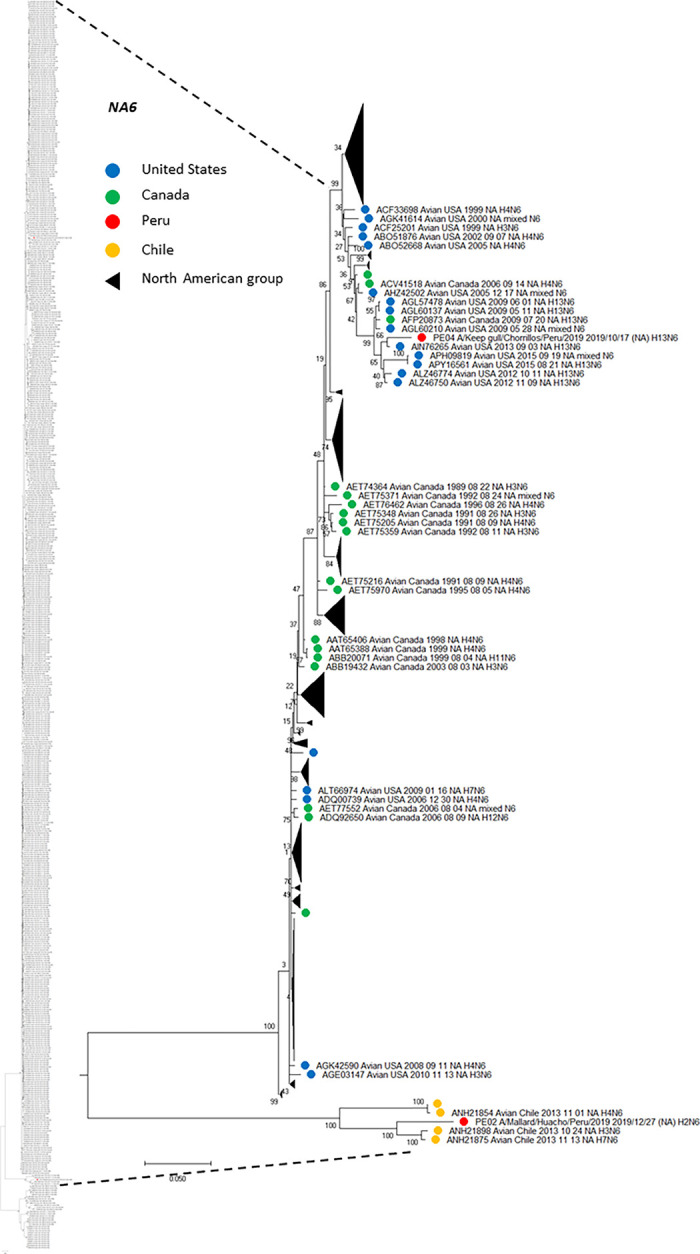
Phylogenetic tree of NA subtype N6 of two isolates detected in a kelp gull and a mallard in Peru, and others detected in America. The tree was generated using a maximum likelihood method with 1000 replicates of bootstrap using GTR+G+I as nucleotide substitution model. They were included in the tree with the Peruvian strains (PE-02 and PE-04) and 585 sequences of complete coding region of NA subtype N6 that was detected in America through all time. A subtree of the complete phylogenetic analysis, including isolates identified in our study is shown. N6 sequences from United States (blue), Canada (green), Chile (orange) and Peru (red) are deployed. Collapsed groups of North American N6 sequences are shown in black triangles. The complete phylogenetic tree is shown at the side for reference.

**Fig 6:**
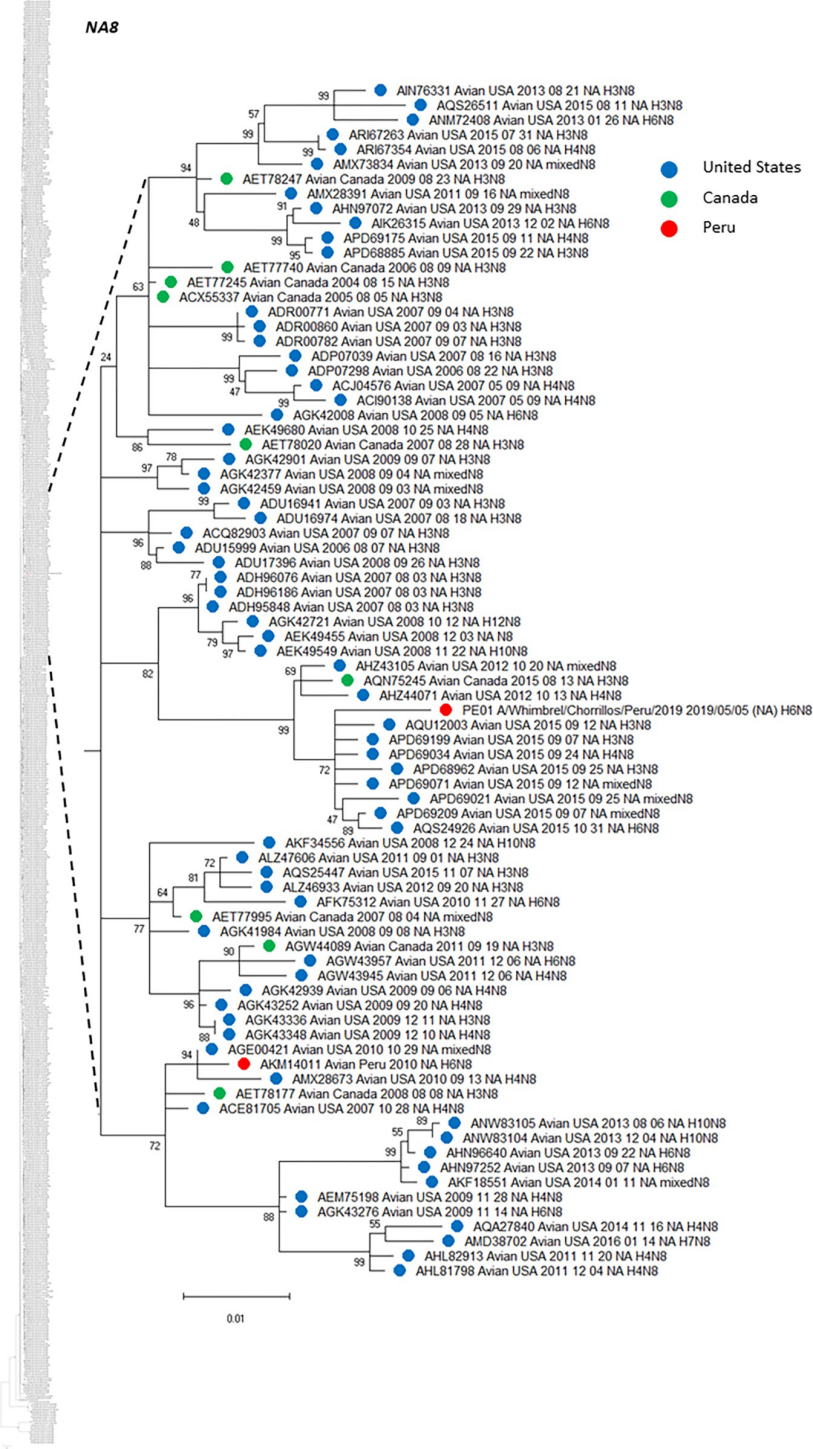
Phylogenetic tree of NA subtype N8 of an isolate from a whimbrel in Peru and others detected in America. The tree was generated using a maximum likelihood method with 1000 replicates of bootstrap using GTR+G+I as nucleotide substitution model. They were included in the tree with the Peruvian strain (PE-01) and 741 sequences of complete coding region of NA subtype N8 that was detected in America through all time. A subtree of the complete phylogenetic analysis, including the isolate identified in our study is shown. N8 sequences from United States (blue), Canada (green) and Peru (red) are deployed. The complete phylogenetic tree is shown at the side for reference.

Based on NA sequence analysis, we also deployed phylogenetic trees including that NA genetic information from virus subtypes that have been circulating in America. Similar to HA analysis, Figs 4 (N2), 5 (N6) and 6 (N8) show the phylogenetic relationship among NA genes reported to date, presented as subtree versions of complete phylogenetic trees. Hence, we identified that both N2 from our two H6N2 isolates arose from two divergent ancestors. N2 (PE-05) presented the highest similarity with a strain H5N2 (US/Arkansas/2015), with 99.56% of amino acid identity, while N2 (PE-03), clustered with three different strains from Chile in 2016, presenting 98.69% amino acid identity with the closest relative. Furthermore, the phylogenetic tree showed that the both N2, isolated in the same site from Peru (77.1595302°W, 12.0717358°S), from the same wild bird species (Franklin’s gull), sampled one week apart (13^th^ and 19^th^ of December, 2019), grouped in two different clades. For N2 (PE-05), it grouped with all US isolates. The other strain (PE-03) exhibited the closest relationship with those identified in Chile and Argentina (Fig 4). In terms of N6, the one from PE-02 had the highest similarity with a strain from Chile in 2017 (Chile/2017, H7N6), whereas, the N6 sequence from our H13N6 isolate was closely related to the US isolate (US/Minnesota/2017/H13N6) with 99.57% amino acid identity. The phylogenetic tree presented the topology with the same characteristics (Fig 5), showing that Peruvian H13 strain formed a monophyletic group with Chilean strains identified in 2013 (H3N6, ANH21898 and H7N6, ANH21875) and the other, with strains from US also identified in 2013 (four H13N6 isolates, AIN76265, APY16561, ALZ46774, ALZ46750 and a mixed N6, APH09819). Moreover, N8 from PE-04 had more similarity with the US isolate (US/New Jersey/2017/H5N8) having 99.57% amino acid identity (Fig 6).

## Discussion

To conduct surveillance of avian influenza A virus (AIV) circulating among the wild birds in Peru, we collected and tested fecal samples from several species of birds along the coast of Peru. We isolated five AIVs and their whole genome was sequenced. Genetic analysis of HA and NA genes revealed that these viruses are of subtypes H6N8, H13N6, H6N2 and H2N6. Our results also demonstrated a high HA and NA variability in our isolates, as compared to others previously identified, indicating the active viral circulation within the populations assessed. Despite the multiple orders evaluated, *Anseriformes* and *Charadriiformes* orders are the main natural reservoir of AIV in Peru. According to Rejmanek et al. (2015) [28, 29], H6N2 and H6N8 have been frequently isolated from mallard and goose (*Anseriformes*), while H13 and H16 have been exclusively isolated from gulls (*Charadriiformes*). Our findings show that this pattern varies, which might explain in part, the higher variability of HA and NA observed. Further studies are required to assess the implications of host variation on viral evolution. Nevertheless, all isolates found in the study belonged to subtypes commonly known as low pathogenic avian influenza viruses (LPAIV).

The HA protein present 10^−4^ amino acid substitutions / site / year under action of purifying selection, mainly driven by the host immune responses [30]. This suggests that viral genetic structure tends to be determined by “space”, “territoriality” under a sympatric speciation model, indicating that evolutionary events within the same migration route are more likely to occur [31]. In our study, sites where we detected those AIV isolates are common geographical sites for migratory birds feeding and resting within their migratory route, considering that these places create potential sites for emergence of novel strains. On the other hand, according to HA specificity in the host cell receptor, each AIV subtype has a group of susceptible animals to infections, and in multiple cases, include humans in the center of this circulation pattern [1]. In this context, public policy actions must consider a one-health dynamics, in which all factors must be considered, from factors such as environmental preservation, animal and human health, since only this perspective will have the possibility of success in regional and global epidemiological surveillance for emergency containment of highly pathogenic strains.

**Fig 7:**
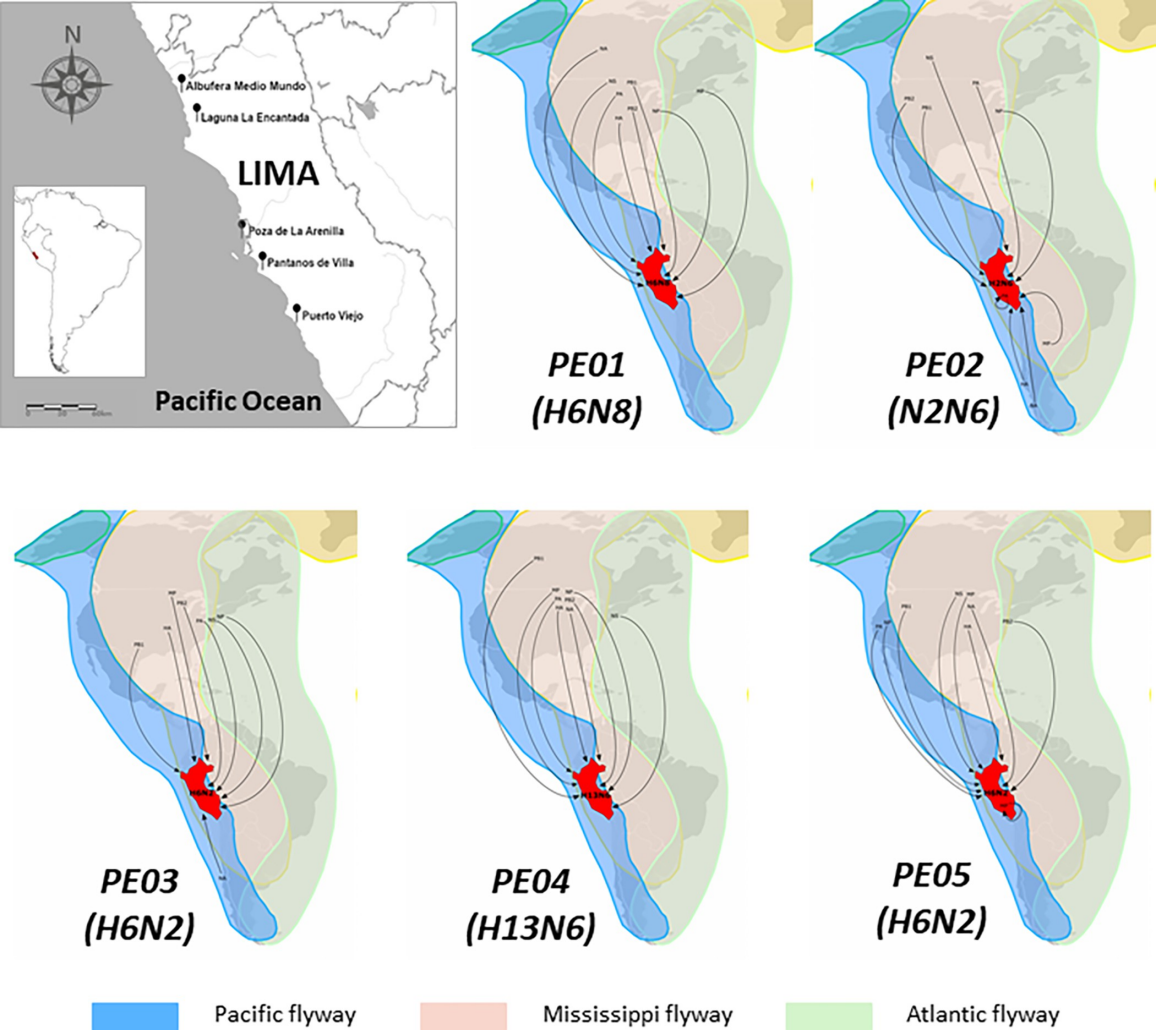
Schematic representation of genetic flow of avian influenza virus isolates obtained in the study. Most of the viral genes has closely related relatives from isolates in North America, reflecting the genetic flow of AIV isolated in Peru comes from relatives in North American countries. Isolates PE-02 (H2N6) and PE-03 (H6N2) has both NA genes with common relatives from South America. In addition, sample PE-02 has also HA and MP from common ancestor from Chile and Argentina, respectively. PE-05 has a MP gene closely related to an isolate from Peru. The Pacific (sky blue), Mississippi (orange) and the Atlantic (green) flyways are shown.

Peru is a biodiverse country for multiple avian species and a special location to identify new strains of AIV since it is located right at the crossroads of Pacific and Mississippi migratory routes. Our study identified five different isolates, including four subtypes obtained from four different migratory bird species. Our isolates presented hemagglutinin whose subtypes originated from US (H2, H6 and H13 presented closest relationship with isolates identified in US and Canada); while the neuraminidase of our isolates originated from Chile (N2), Chile and US (N6). Since our isolates were obtained from migratory birds, these findings suggest that Peru, with its geographic position is an important site for emergence of novel reassortants of AIV. A schematic representation of the genetic flow across Americas involved in the evolution of AIV in Peru is shown in Fig 7. The H2 identified in a mallard presented a special attention since it could be transmitted to humans directly or through domestic poultry infections and possibly associated to cause the next flu pandemic [32, 33]. However, little is known in terms of viral interaction with its natural host. AIV H2 produced a severe pandemic in 1957 and was reported circulating in North America in the end of 90’s [34]. On the other hand, the H6 was identified in two different species, in a whimbrel and in a Franklins’ gull. Interestingly, domestic poultry has been reported to be extremely susceptible to this subtype [35], representing an economic and social threat. H6 segment has been intensely identified in mallard from Canada and presents global distribution. Moreover, H6N2 and H6N8 subtypes have been reported in several parts of America for more than forty years. For instance, H6N2 was identified in USA (1986–2015), in Mexico (2007) and Argentina (2010) and Chile (2016–2017) [36]. On the other hand, H6N8 has been circulating in America between 1977–2016 in USA, detected in Argentina and Peru in 2021; and in Chile, in 2016 and 2017. Thus, H6N8 is described as a high-priority subtype [29]. Similar to H2 subtype, H6 has been used experimentally in ferrets to produce vaccine candidates [37]. Finally, the H13 identified in a kelp gull can be spread to domestic poultry and other mammals. Interestingly, H2 and H13 have shown global distribution, while H2 subtype has a closest phylogenetic relationship with H5, indicating a more recent differentiation between these subtypes. This close phylogenetic relationship between H2 and H5 might induce events for viral reassortments.

In regard to NA, N2 subtype isolated from two Franklin’s gulls has shown to infect swine, marine mammals, dogs, poultry, and humans. For instance, H9N2 subtype has presented high prevalence in domestic and wild birds and has been isolated from swine and humans, which has strong ability for inter-species transmission [38]. N6 has also been described as a high-priority subtype [29] and was identified in two different strains in our study, one obtained from an *Anseriformes* (mallard) and other from a *Charadriiformes* (kelp gull). This N6 subtype needs special attention because in addition to infecting aquatic mammals, it can infect swine and thus suitable for genetic changes [39]. Finally, N8 subtype has been widely identified in America, sometimes in association with the subtype H6, as we found in our study, and is considered as a high-priority subtype [29].

Some evidence highlights that strains identified in the same flyway are more similar between them than those found in others flyway during a period [32]. The America continent presents three different flyways: the Pacific, the Mississippi and the Atlantic. In our study, samples were obtained from animals from the Pacific flyway. Further studies are required to assess the viral circulation in flyways crossing other areas in South America. Hence, considering the spatial evolution, AIV strains seem to be spatially structured. Nevertheless, in multiples places, the overlapping of the flyways facilitates the genic flow and the possibility to generate novel strains. Additionally, in the northern region such as Canada and Alaska, there is a confluence of multiple flyways from several parts around the world and thus AIV strains do not show spatial genetic structure [40]. Finally, our results indicate that multiple AIV subtypes are circulating in wild birds in Peru. Even though those LPAIV subtypes have been previously detected, this work has revealed that these isolates possess unique features at the nucleotide and protein level. Although we have not defined what the implications of these amino acid changes represent on HA and NA function, our results provide novel insights into AIV evolution for genetic surveillance.

## Conclusion

Our study shows the active circulation of multiple AIV subtypes in wild birds in Peru. Despite their close genetic relationship with other common subtypes previously found in America, our findings have identified that these isolates have gained multiple unique changes during the viral evolution in America. Altogether, this work has shed the light on the importance of Peru, which confers ecological advantages as relevant geographic location for the emergence of novel AIV in America with pandemic risk.

## Supporting information

S1 Fig PCR performed using specific primers for NP segment of influenza virus type A described by Wright et al., 1995. Isolated Peruvian avian influenza viruses showed a 306bp band. M: 100bp molecular marker (Applied Biological Materials Inc.); lane 1, 2, 3, 4, 5: Peruvian AIV isolates; lane 6, 7, 8, negative samples; NC: negative control, Newcastle Disease Virus; PC: positive control, Avian Influenza Virus; NTC: No template control. Electrophoresis performed on 1.5% agarose gel and 1X TAE buffer.
(TIF)
