# Supplementary figures and images for: Genetic subtyping and phylogenetic analysis of HA and NA from avian influenza virus in wild birds from Peru reveals unique features among circulating strains in America
Source: PLoS One. 2022 Jun 7;17(6):e0268957. doi: 10.1371/journal.pone.0268957 (PMC9173603; doi:10.1371/journal.pone.0268957)

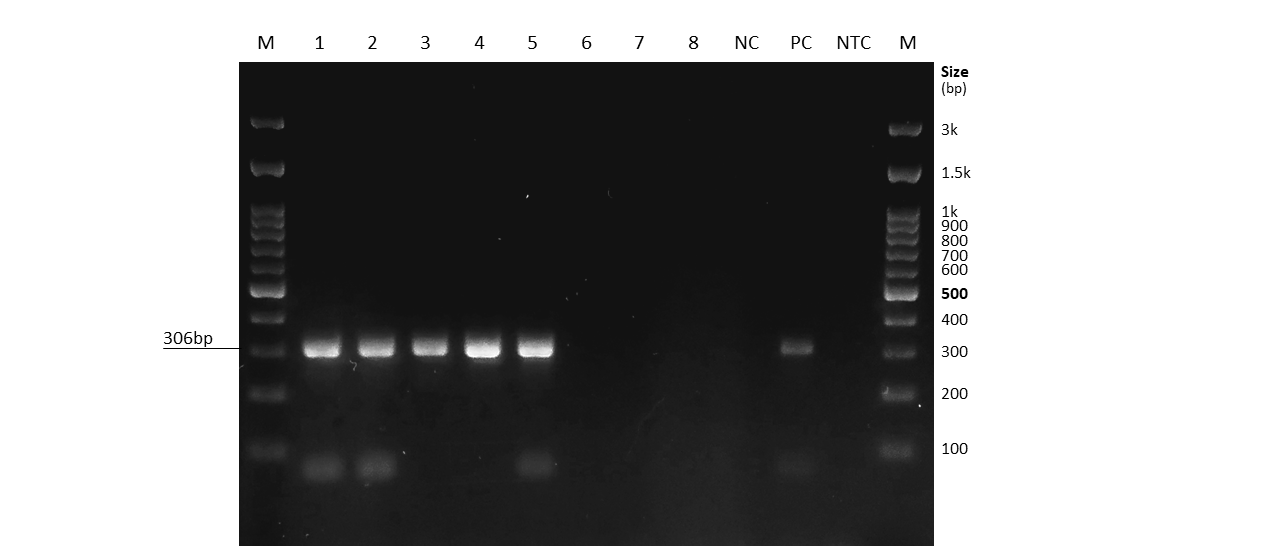

Supplement: S1 Fig — Isolated Peruvian avian influenza viruses showed a 306bp band. M: 100bp molecular marker (Applied Biological Materials Inc.); lane 1, 2, 3, 4, 5: Peruvian AIV isolates; lane 6, 7, 8, negative samples; NC: negative control, Newcastle Disease Virus; PC: positive control, Avian Influenza Virus; NTC: No template control. Electrophoresis performed on 1.5% agarose gel and 1X TAE buffer. (TIF) [file pone.0268957.s001.tif]
